# Charcot spinal arthropathy: a case series

**DOI:** 10.1038/s41394-025-00709-x

**Published:** 2025-05-23

**Authors:** Vanessa Jane Chow, Akter Hossain, Maurizio Belci, Shyam S. Swarna

**Affiliations:** 1 National Spinal Injuries Centre, Stoke Mandeville Hospital, Buckinghamshire NHS Foundation Trust, Aylesbury, UK; 2 University of Cardiff, Cardiff, Wales UK

**Keywords:** Trauma, Diagnosis, Orthopaedics

## Abstract

**Study design:**

A retrospective case series analysing six cases of Charcot spinal arthropathy.

**Objectives:**

To evaluate the etiology, clinical manifestations (e.g., pain, trunk instability, autonomic dysreflexia, spasticity), latency period before symptom onset, affected spinal regions, and treatment strategies for Charcot spinal arthropathy.

**Setting:**

National Spinal Injury Centre, United Kingdom.

**Methods:**

Data were collected and analysed from six patients diagnosed with Charcot spinal arthropathy. Variables examined included the etiology, clinical presentations, spinal regions affected, latency period, and outcomes of treatment approaches. Both conservative management and surgical intervention strategies were evaluated.

**Results:**

Patients exhibited common clinical manifestations such as pain, loss of trunk control, autonomic dysreflexia, and spasticity, with varying latency periods before symptom onset. The thoracic spine was the most frequently affected region. Conservative management successfully stabilized symptoms in most cases, while surgical intervention was necessary in instances of severe trunk instability, refractory pain, or deformity impacting mobility.

**Conclusion:**

Conservative management should be the initial treatment approach for Charcot spinal arthropathy. Surgical intervention is reserved for cases with significant clinical progression, such as unresolved pain, mobility restrictions due to trunk deformity, or urgent complications arising from spinal pathology.

## Introduction

Charcot spinal arthropathy (CSA), also referred to as spinal neuroarthropathy or neuropathic spinal arthropathy, is a rare yet progressive osseous and ligamentous injury leading to the deterioration of vertebral joints and significant morbidity including chronic pain, instability of the spine, deformity, and severe neurological deficits. This disorder arises in situations where there is reduction in afferent nerve signals, resulting in the diminished sensation of deep pain and proprioception within the spinal column [1]. Accurately determining the prevalence of CSA is challenging due to the limited data primarily derived from case reports and series. However, it is estimated that CSA affects approximately one in every 220 patients [2]. This estimation is based on the context of around 4400 new cases of spinal cord injury (SCI) each year in the UK and an estimated 105,000 individuals currently living with SCI in the country [3].

Initially identified in 1978 as a sequalae of traumatic SCI, the preponderance of CSA cases has been recognized as a rare long-term complication of SCI [4]. As the life expectancy of individuals within this patient cohort extends, and as the frequency of surgical spinal interventions increases, it is anticipated that spine specialists will encounter Charcot spine with increasing regularity in the future. An enhanced comprehension of its pathophysiology, along with a deeper insight into the altered biomechanics and bone metabolism in the context of their clinical presentation and complications associated with CSA, is imperative for the provision of optimal individualised patient care.

Within this framework, the purpose of this retrospective case series is to articulate our experiences, highlight the challenges encountered, and delineate the management strategies employed for Charcot spinal arthropathy in a rehabilitation center, thereby contributing to the body of existing knowledge and potentially guiding future clinical practice in this specialised area.

## Methods

This investigation was undertaken at the National Spinal Injury Centre in the United Kingdom, a distinguished rehabilitation facility renowned for its specialisation within the country. A comprehensive search of the database was conducted by the educational department with the aim of identifying patients diagnosed with spinal neuropathy, neuropathy, and Charcot spine. Subsequently, an Excel spreadsheet containing the initial search results was meticulously examined by two independent reviewers to extract data on patients who met the predefined inclusion criteria: (1) patients who were treated at our unit and (2) diagnosed with CSA. Through a systematic retrospective analysis, a total of seven cases of CSA were identified, diagnosed, and treated within our institution, encompassing the period extending up to March 2024, for which data were accessible. However, one patient who initially provided consent later withdrew it and was consequently excluded from this case series. This case series offers an in-depth exploration of the occurrence, disease trajectory, clinical manifestations, radiological findings, and outcomes of treatment pertaining to CSA.

## Results

We report on six clinical cases of Charcot spine in patients who had spinal cord injury. One hundred percent of the patients were male, the mean age of initial injury leading to their SCI was 21.17 ± 12.09 years (range: 2–42) with an average time lag between the index injury and the diagnosis of CSA of 37.17 ± 11.29 years (range: 20–52). All patients exhibited the development of CSA within the lumbar region. The demographic data is presented in Table 1, while CSA related findings are detailed in Table 2. These cases were rigorously evaluated within a multidisciplinary team (MDT) setting, involving radiological assessment and consultation with spinal surgery experts, to ensure diagnostic accuracy.

**Table 1:** Patient demographics and initial spinal cord injury characteristics.

Patient	Gender	PMH	ASIA grade	Age of accident (years)	Cause of Injury	Surgical Treatment of SCI
1	Male	Heterotrophic Ossification Right syringopleural shunt insertion Active man	T4 AIS A	18	RTA	Posterior rod and screw fixation from T2 – L2
2	Male	HTN, OA Rt TKR R shoulder arthroscopy Prv sphincterotomy for his bladder	T10 AIS A	28	RTA	Harrington rods from T11 to L4
3	Male	Femorectomy in 2007 Left shoulder subacromial arthroscopy and decompression 2001	C6 AIS A	20	RTA	C6/7 Decompression laminectomy
4	Male	HTN Renal transplant in 2007 – on immunosuppresents AV fistula L thrombosed Bladder removed – ileal conduit formation Bilateral parathyroidectomy Previous R girdlestone ORIF L femur Infraspinatus reconstruction	T10 AIS A	2	RTA	Scoliosis correction surgery at age 6 years old T8 pelvis stabilisation at the age of 18
5	Male	Active man Dislocation of left elbow 2008 DVT Duodenal ulcer Spinal abscess (discitis) T5–6	T3 AIS A	17	RTA	Nil surgical intervention for initial SCI
6	Male	Congenital pain insensitivity Left BKA at age 12 secondary to bone deformity Right leg deformity due to previous fractures Multiple right Ankle fractures Right humerus fractures Rheumatoid arthritis	T9 AIS A	42	Microfractures from years of traumatic injuries due to congenital pain insensitivity syndrome	L1/L2 Spinal Abscess drainage under CT guidance

**Table 2:** Details of charcot spinal arthropathy in patients, covering clinical presentation and treatment modalities.

Patient	Level of CSA	Time lag between SCI and CSA diagnosis (years)	Age of CSA diagnosis (years)	Clinical presentation	Treatment of CSA	Complications noted
1	L3/L4	25	43	Autonomic dysreflexia Lower limb spasms Localised back pain Sitting imbalance Spinal deformity	Steroid injection at the charcot joint Pelvic fixation offered Rehabilitation (including Allied Health Care)	Nil documented
2	L3/L4	42	70	Progressive lumbar pain for nine months Feeling of sinking forward Loss of height Esophageal reflux Increasing disability and distress	Harrington rods removed Fixation of lumbar Charcot – posterior instrumented correction and fusion to pelvis Rehabilitation (including Allied Health Care)	2 days ITU admission, Day 7 Pulmonary Embolism
3	L4/L5	52	72	Autonomic Dysreflexia Hypotensive episodes Loss of consciousness Incidental Finding	Conservative (analgesics, antispasmodics) Rehabilitation (including Allied Health Care)	Nil documented
4	L4/L5	46	48	Back pain Unpleasant sensation/dull ache around left pelvis and hip Crunching and grinding in the lower lumbar spine Deterioration in his seating posture – now slumped over	Scoliosis correction surgery at age 6 years old Revision decompression and fusion at L4/5, osteotomy performed at this level and instrumentation down the pelvis Rehabilitation (including Allied Health Care)	1 year follow up found decreased hip pain, back pain, leg pain, experiences mild clinic around spine on long periods of sitting in chair. 2 year follow up was satisfactory
5	T10/11	38	55	Progressive lower back pain Clicking and crunching of his back Loss in height Spinal deformity Left leg spasms	TLSO Brace Posterior deformity correction of kyphosis and thoracolumbar fusion of unstable Charcot joint Rehabilitation (including Allied Health Care)	ITU admission post op for 2 days
6	C7–T1 and L2/L3	20	62	Scoliosis Autonomic Dysreflexia	Conservative (analgesics, antispasmodics) Rehabilitation (including Allied Health Care)	Nil documented

### Patient 1

**Fig. 1:**
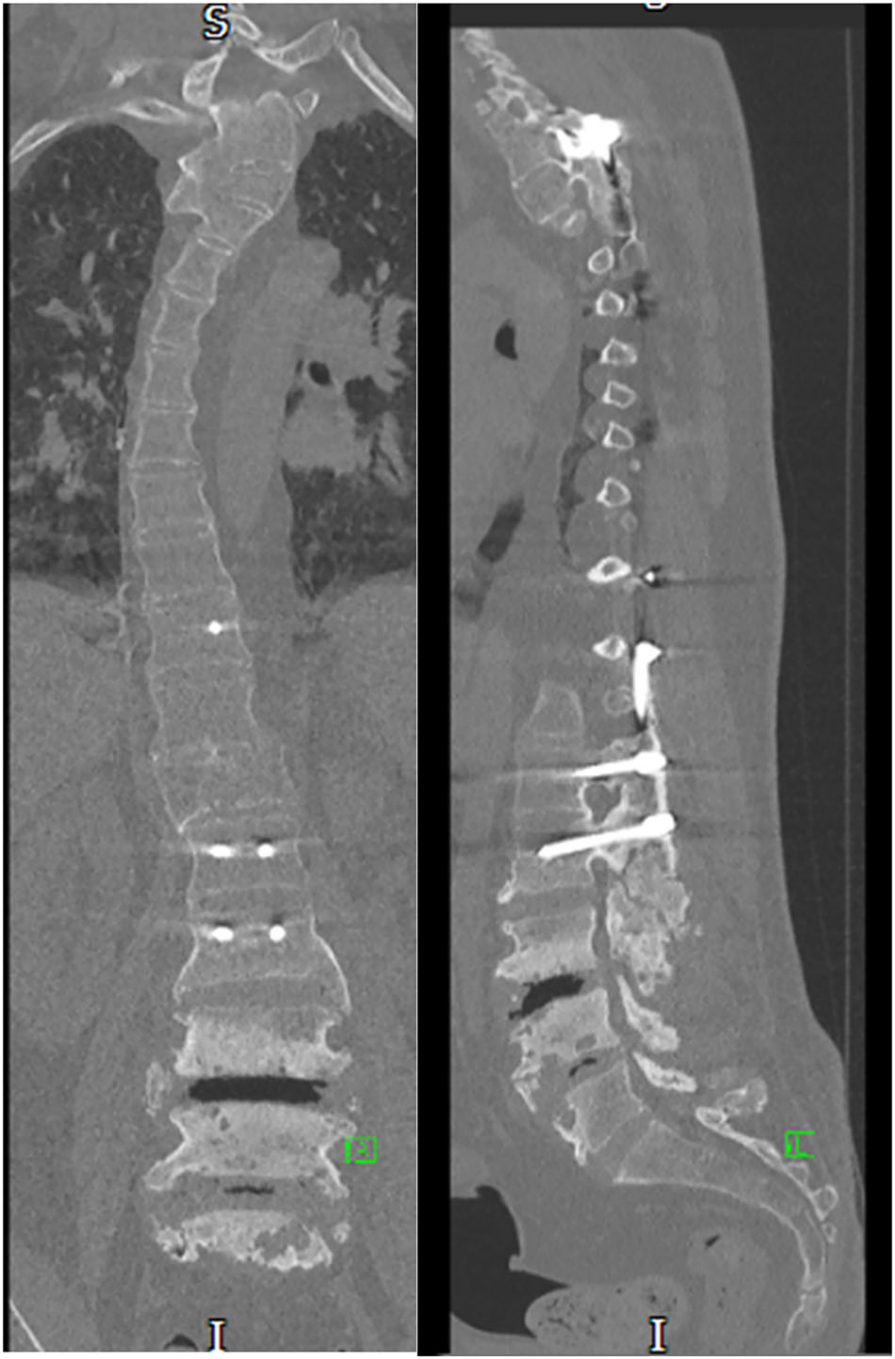
Coronal and sagittal view of the thoracic and lumbar spine CT: posterior rod and screw fixation noted from T2–L2. There is impression of solid fusion through these levels. Vacuum phenomenon and bony overgrowth noted at L3/L4.

The initial case featured a male patient who sustained a spinal cord injury at the T4 level, classified as AIS A on the American Spinal Injury Association (ASIA) Impairment Scale, as a result of a road traffic accident at the age of 18. At that time, he underwent posterior stabilisation spanning T2 to L2. The development and diagnosis of his Charcot joint occurred at age 43, localised at L3/L4, below his initial fixation (Fig. 1).

Initially, this was managed conservatively, treated for pain and spasms. However, he was admitted to our facility a few years after due to progressive back pain, spasms and recurrent autonomic dysreflexia (AD), coupled with worsening of spinal deformity, and impaired sitting balance whilst in his wheelchair which was especially distressing due to his active lifestyle. To alleviate his back pain, a corticosteroid injection was administered directly into the affected joint. Although he experienced temporary relief, the patient’s pain recurred shortly thereafter. Upon discharge, he was presented with the option of undergoing spino - pelvic fixation to address his ongoing symptoms.

### Patient 2

**Fig. 2:**
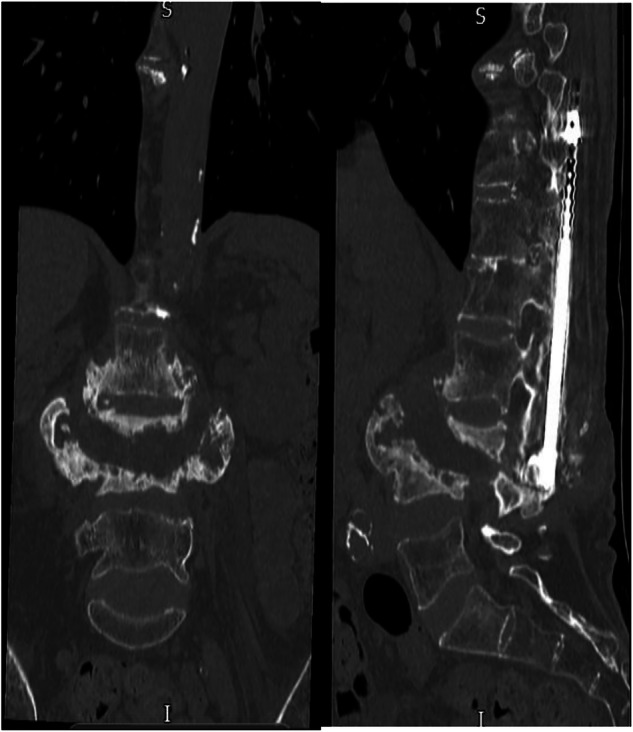
Coronal and sagittal view of the thoracic and lumbar spine CT: anterior translation of the inferior half of the L4 vertebral body in relation to the superior endplate extensive destructive/proliferative changes within L4 with diffusely sclerotic vertebrae associated erosive changes. Extensive anterior and marginal hypertrophic bone formation is noted. The ex vacuo phenomenon seen.

We present the case of a male patient who sustained a traumatic SCI at the age of 28, leading to complete paraplegia. The injury, which was a consequence of a road traffic accident, was classified at the neurological level of T8 AIS A. Following this, he promptly underwent surgical stabilisation with Harrington rods from T11 to L4. Forty-one years post-accident at the age of 70 years old, he reported to the spinal clinic, experiencing progressive lumbar spine pain over the last nine months, coupled with new sensation of forward sinking, loss of height, and significant discomfort, leading to increased disability and distress. Further investigations confirmed findings consistent with CSA at the L3/L4 level (Fig. 2).

**Fig. 3:**
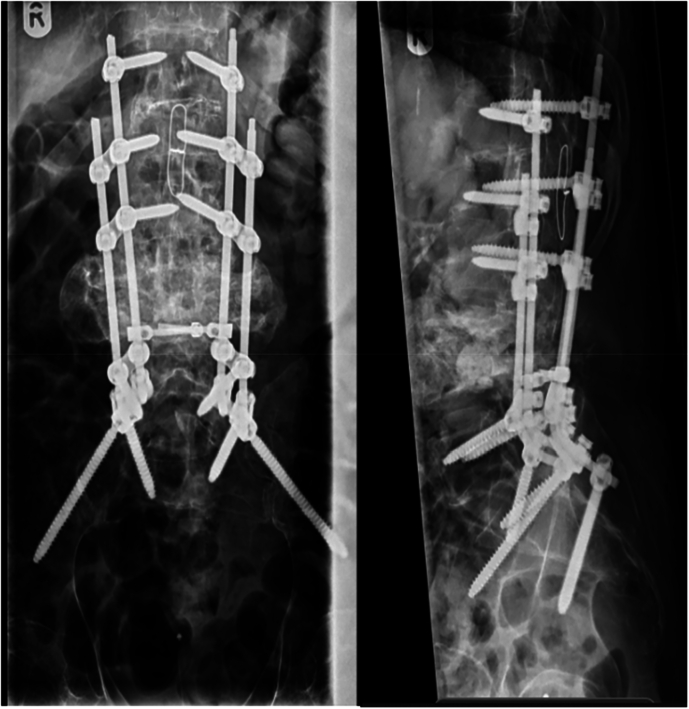
Coronal and sagittal view of the thoracic and lumbar spine XR. Extensive bilateral lumbosacral posterior spinal instrumentation bridging the Charcot’s spine at L3/L4 in situ.

After a thorough consultation process, a decision was made to proceed with surgical intervention, which included the removal of the previously placed Harrington rods and the implementation of fixation for lumbar Charcot via posterior instrumented correction and fusion to the pelvis. The procedure involved the insertion of pedicle screws into the three intact vertebrae above the Charcot site, at L5, S1, and through pelvic iliac screws (Fig. 3). The Charcot lesion, which had resulted in a complete dissociation and a 4 cm anterior translation of L5, was fully addressed, with fusion achieved across the vertebrae situated above the Charcot site. Having been followed up for two years, there has been no complications related to the surgical intervention.

### Patient 3

**Fig. 4:**
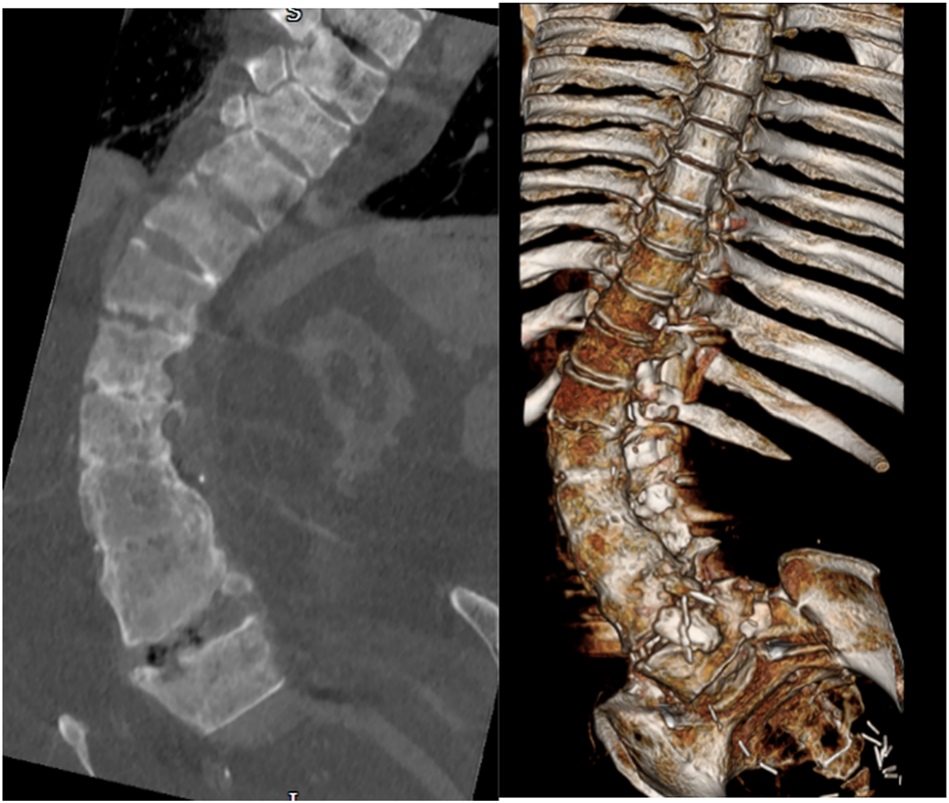
Coronal view of thoracic and lumbar spinal CT: a break in the fusion mass at the L4/L5 level is noted, where there is vacuum phenomenon and bony irregularity within the disc space locally. There is also a mature appearing cleft/deficit within the posterior elements at this level. The appearances locally are suggestive of a pseudoarthrosis secondary to failure at the lower end of the effusion. Bony overgrowth is leading to modelling abnormality and canal deformity.

**Fig. 5:**
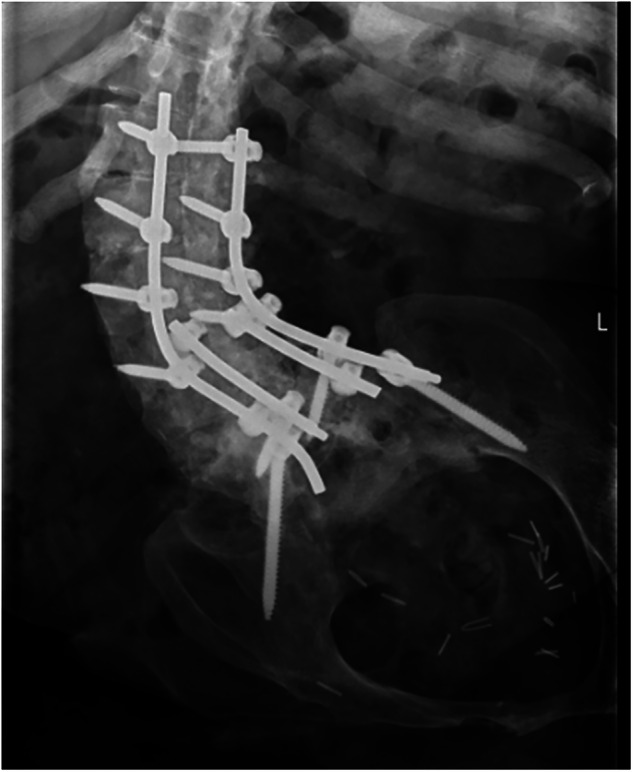
Thoracic spine XR. Evident scoliosis and a long thoracolumbar fixation.

Here, we document a male patient, who sustained a traumatic spinal injury from a road traffic accident at the age of 20, subsequently undergoing a decompression laminectomy at C6/7. On admission, his injury was classified as C6 AIS A. Fifty-two years post-injury, at the age of 72, he received a diagnosis of CSA at the L4/5 level following an incidental discovery on CT imaging during his presentation to the spinal unit (Fig. 4, Fig. 5). His presentation was characterised by alternating recurrent episodes of AD, succeeded by episodes of hypotension that culminated in loss of consciousness. His paroxysmal hypotension and autonomic dysreflexia was found to not be caused by his CSA but due to issues with the Intrathecal Baclofen pump which he had implanted previously for ongoing spasticity. Addressing the ITB issues, resolved his symptoms. He required no surgical interventions pertaining to his Charcot joint due to its early state and low burden of his current situation. The incidental identification of his CSA at an early stage presents an advantageous opportunity for timely monitoring and management. Consequently, this condition will be systematically evaluated on an annual basis during his scheduled routine follow-ups, ensuring comprehensive oversight and intervention.

### Patient 4

**Fig. 6:**
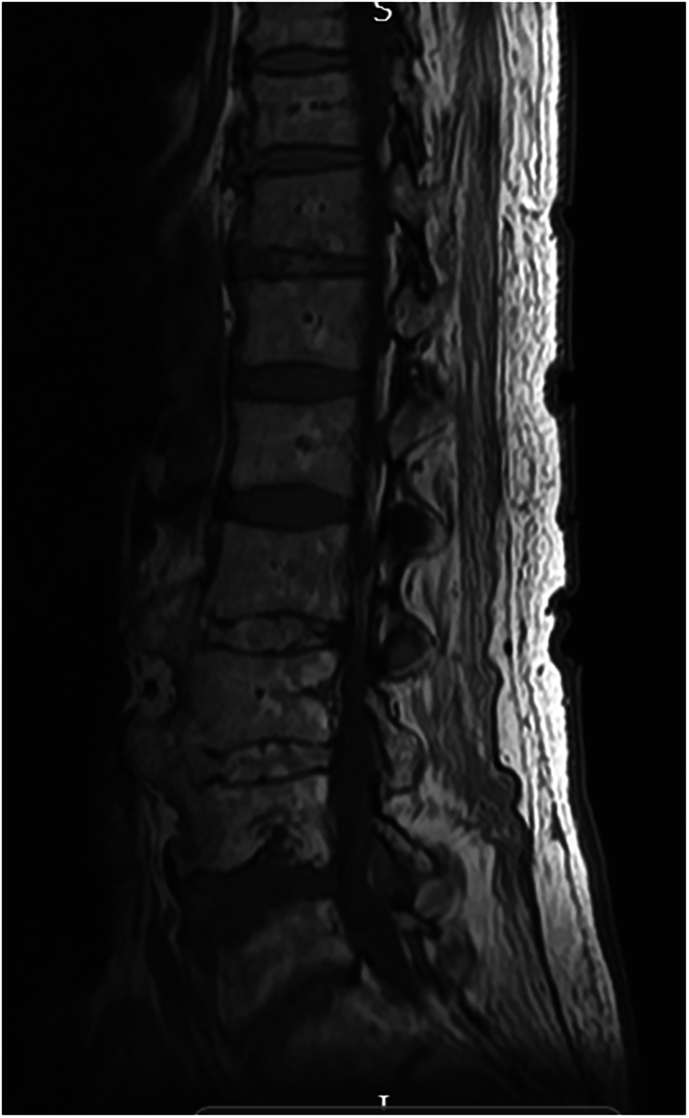
Sagittal view of the lumbar spine CT. Widening of the L4 and L5 intervertebral disc spaces with associated fluid signal and endplate irregularity, appearances may represent early Charcot’s spine.

Our next male patient experienced a traumatic spinal cord injury at the age of two, following involvement in a road traffic accident. This was classified with a neurological level of T10 AIS A. In addressing spinal deformities resultant from his injury, he underwent scoliosis corrective surgery seven years post-accident. Remarkably, 46 years following the initial injury, the patient presented at the clinic with progressive pain and discomfort, along with a crunching and clicking sensation when sitting in his wheelchair. He was diagnosed with Charcot spinal arthropathy at the L4/5 level and subsequently underwent a revision decompression and fusion procedure at this level (Fig. 6). Over the next two years of follow-up, he demonstrated satisfactory clinical improvement.

### Patient 5

**Fig. 7:**
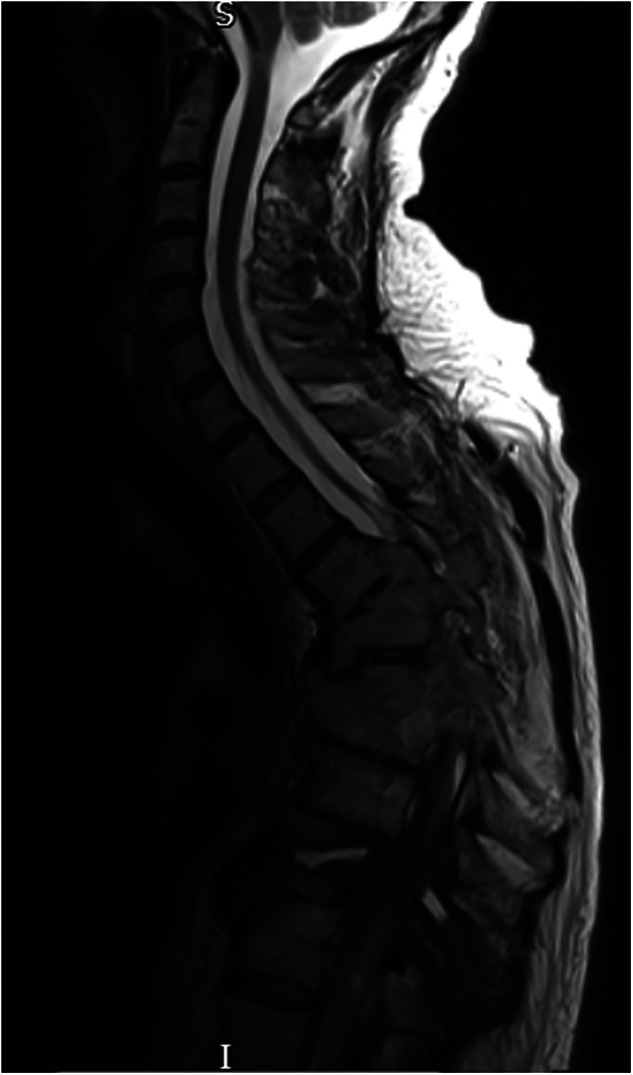
Sagittal view of the cervical and thoracic spine CT: there is evidence of chronic injury and post-traumatic fusion of T3, T4, T5, T6, T7 and T8. At the inferior aspect of this fused segment there is destruction of the T11 vertebral body with a fluid filled cleft extending through all three columns consistent with Charcot spine formation. There is no local marrow oedema within the T12, T10 and T9 vertebral bodies. There is severe cord compression at the level of T10 Charcot pseudoarthrosis and hyperintense signal within the distal cord and conus.

**Fig. 8:**
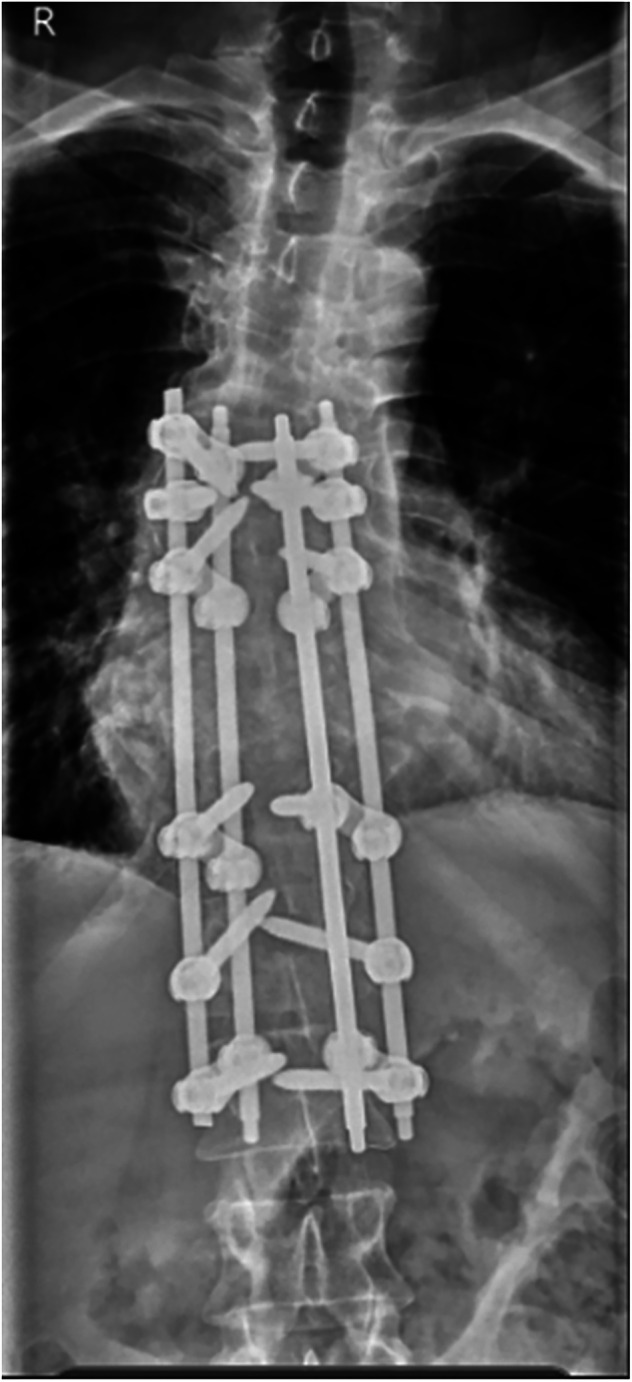
Lumbar spine XR. Spine thoracic fixation across the T10/11 Charcot spine.

A 17-year-old gentleman sustained a traumatic spinal cord injury from a RTA, with the neurological level of injury T3 AIS A. He maintained an active lifestyle. Thirty eight years following his injury he presented to the clinic with complaints of progressive lower back pain, clicking and crunching of his back, loss of height and left leg abductor spasms. Additionally, he developed further spine deformity, notably kyphosis. At this point his MRI scan confirmed advanced Charcot in T10/11 (Fig. 7). He was treated with a TLSO brace and eventually underwent posterior deformity correction of kyphosis and thoracolumbar fusion for his unstable charcot joint (Fig. 8). Post-surgery, he gradually resumed his regular bowel management and transfer routines, extended the time spent in the car, returned to using the standard toilet, and aimed to transition from sleeping in the medical bed downstairs to his own bed again.

### Patient 6

The final patient in this case series, a male born with congenital pain insensitivity (CIP), suffered multiple fractures and injuries to his lower limbs, culminating in a below-knee amputation of his left leg. It was noted that due to his inability to sense pain, he would frequently jump from very high places. Despite these challenges, he continued to walk using a prosthesis for his left leg. However, at the age of 42, he began experiencing weakness in both lower limbs. Subsequent investigations revealed significant spinal column injuries. He was hospitalised for six months, whereby he became incontinent and suffered immense pain. He underwent multiple level decompressions to address spinal central stenosis and received treatment for an infection in his spinal column. Upon discharge, he was mobilising with crutches and had regained full function of his bowels and bladder. Subsequently, he experienced progressively worsening weakness in his lower limbs, necessitating several decompression surgeries, along with washout and debridement procedures, over a span of six years. When he was 59, one morning, he awoke to find his lower limbs insensate and immobile. Initially treated for urosepsis, further imaging revealed an L1 spinal abscess, which was drained, followed by antibiotic treatment. During this period, he also received treatment for a grade 4 sacral pressure ulcer, which took a year to heal.

**Fig. 9:**
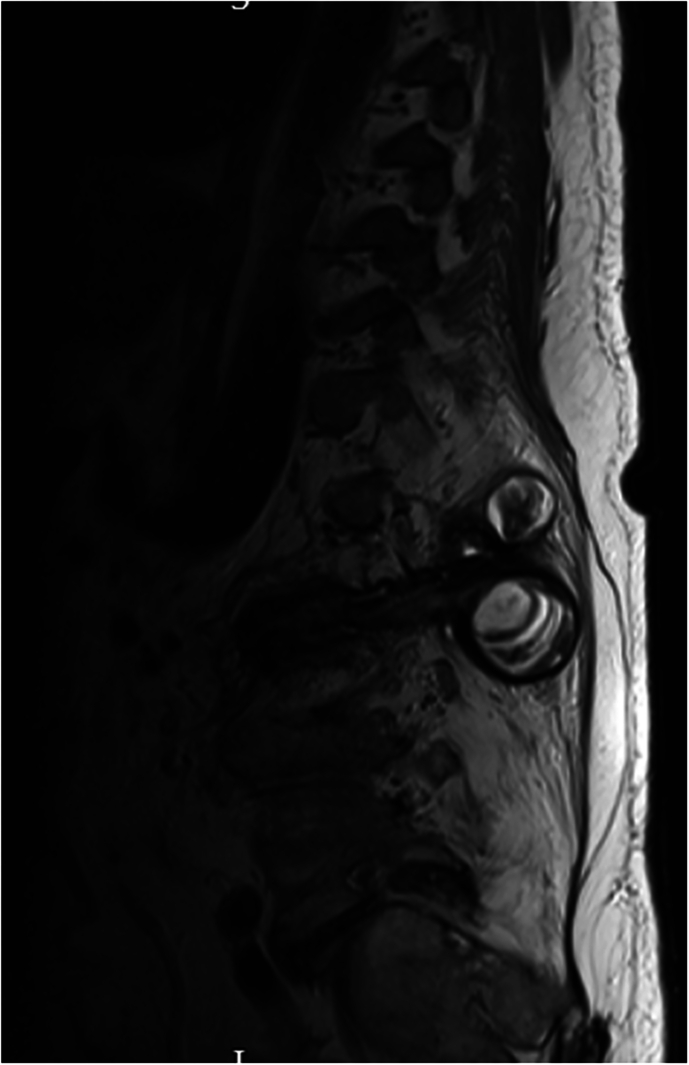
Sagittal view of lumbar MRI: L2/L3 irregular and displaced spinal pseudoarthrosis above the fusion segment. Soft tissue material interposed locally with posterior parents paraspinal fluid collection measuring 7.5 cm.

**Fig. 10:**
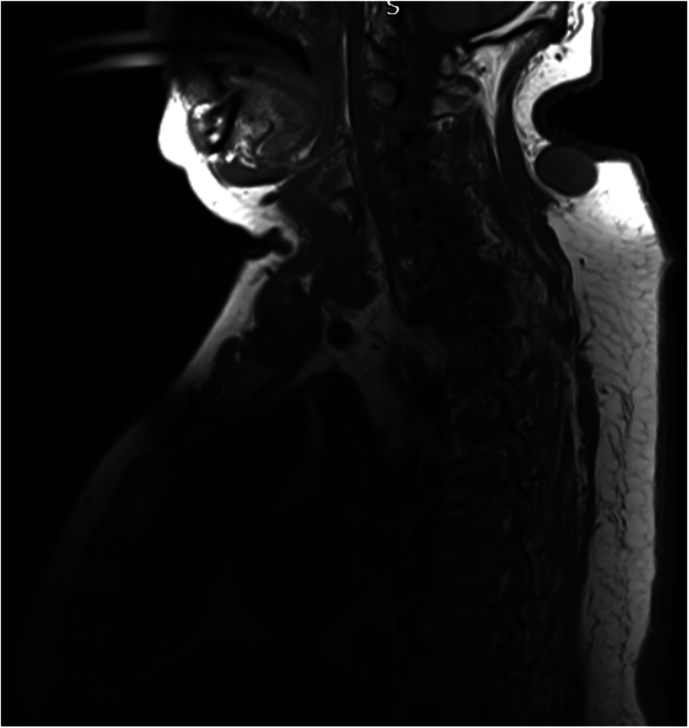
Sagittal view of cervical MRI. Charcot spinal arthropathy noted at the levels of C7-T1 with disc narrowing and edema.

On admission to the rehabilitation unit, his neurological level was classified as C5 AIS A, at the age of 62. He was diagnosed with Charcot spinal arthropathy at the levels of C7-T1 and L2/L3 after admission for recurrent autonomic dysreflexia. This condition was observed above a previously established lower lumbar fusion block segment. CT imaging revealed that at L2/L3, there was evidence of pseudoarthrosis characterized by pronounced bone loss, bony irregularity, and soft tissue interposition, along with bony discontinuity through this segment (Fig. 9). Additionally, disc narrowing at C7–T1 and some degree of oedema were identified, which were either neuropathic or inflammatory in nature (Fig. 10). During a radiological MDT review, both sites were diagnosed as Charcot spine. His Charcot joints have been managed conservatively, given the significant risks associated with spinal surgery and his ability to manage autonomic dysreflexia during his hospital stay. This approach has been supported by educating him on proper sitting positions and the assistance of physiotherapy. He remains stable since.

## Discussion

CSA is a multifactorial, progressive degenerative condition that commonly manifests in the aftermath of traumatic SCI. The pathogenesis of CSA involves the disruption of afferent sensory pathways, resulting in a loss of proprioceptive and nociceptive feedback that normally protects the spine from excessive mechanical loading. This sensory deficit precipitates a cascade of recurrent microtraumatic insults to the vertebral joints, leading to cumulative structural compromise and extensive degeneration. Over time, these iterative injuries culminate in severe joint destruction and spinal instability, underscoring the disorder’s complex and insidious pathogenesis.

### Demographics

Our demographic results align with existing literature, as our patient cohort was exclusively male, with an average age of 21.17 + 12.09 years (range: 2–42) at the initial SCI event. A comprehensive review in 2018, which included 84 patients with 86 Charcot joints, demonstrated a male predominance of 76.1%, and an average onset age of 43.2 years. In this cohort, the L2 vertebra was identified as the most frequently affected site, comprising 29% of the cases, with the L3 and L1 vertebrae closely following at 27.9 and 26.7%, respectively. All patients presented with CSA localized to the thoracolumbar region, and one patient exhibited additional pathology in the cervical spine. The thoracolumbar and lumbosacral regions are particularly susceptible to CSA, given their exposure to substantial biomechanical stress, especially in paraplegic individuals who experience significant mechanical loading during activities such as maintaining an upright posture and executing transfers [5, 6].

### Risk factors

The principal risk factors for developing CSA are conditions that impair spinal proprioception and nociception. In this series, such neuropathic predispositions were exemplified in Patients 2 and 6, who were diagnosed with syringomyelia and CIP, respectively. Notably, Patient 6’s spinal cord pathology was uniquely attributable to CIP—a rare hereditary disorder that eliminates protective pain sensation and joint position awareness. This profound sensory deficit renders individuals particularly susceptible to CSA, wherein progressive, destructive arthropathy of the spine ensues as a consequence of unmodulated mechanical stress and repetitive microtrauma [7]. Affected individuals lack the protective sensation that typically leads to ongoing unrecognized microtrauma, the lack of sensation undermines normal muscular protective responses, facilitating continuous soft tissue damage, ensuing inflammation, and eventual spinal joint destruction and deformity [7]. Staudt et al. (2018), in their comprehensive analysis of CSA among individuals with CIP, noted a conspicuous absence of cervical spine involvement within this cohort, aligning with the general rarity of such cases as evidenced by only a single report in the extant literature [8, 9]. Nevertheless, the diagnosis of Charcot spine in the cervical region of Patient 6 highlights a marked deviation, reflecting profound neuropathic impairment and significant structural compromise. This individual engaged in repeated high-impact activities, such as jumping from heights and landing on their feet, contributing to recurrent cervical spine trauma. Such activities introduce axial loads to the cervical spine, which, in accordance with Newton’s third law, potentially lead to microfractures, ligament strains, or disc injuries due to compressive forces. The neuropathic state alters the typical healing response, triggering unregulated inflammation and osteoclastic activity that results in bone resorption. This cycle of injury and maladaptive healing, compounded by the cervical spine’s intricate biomechanics and reduced load-bearing capacity, increases the risk for developing CSA by perpetuating structural instability and progressive joint destruction.

Traumatic incidents, such as road traffic accidents and falls, were the leading causative factors in this cohort, contributing to repetitive microtrauma and subsequent CSA onset. Surgical interventions like spinal fusions and laminectomies exacerbated the development of CSA. Post-surgical evaluations revealed that regions with the greatest mechanical motion, and therefore increased susceptibility to degeneration, are typically located immediately above or below spinal fixation sites or near laminectomy regions [6, 10].

For instance, extensive spinal fusions spanning multiple vertebral segments introduced significant mechanical stress, which increased the risk of degenerative changes in adjacent vertebrae. Laminectomy procedures further heightened the risk of CSA by destabilizing the posterior spinal elements and compromising the integrity of the paravertebral musculature, leading to an increased reliance on the posterior facet joints and the intervertebral disc-vertebral body articulation for load bearing [11]. This shift in load-bearing dynamics accelerates joint degeneration, particularly in segments already vulnerable to biomechanical stress. Additional factors that intensify joint stress include activities involving lateral bending, torso rotation, participation in high-impact sports, and obesity, all of which impose excessive strain on compromised spinal joints [1, 12]. In individuals with paraplegia, activities such as transferring markedly increase the risk of CSA due to the substantial biomechanical stress exerted on the spinal column during the manoeuvring of the lower extremities and the forceful extension of the triceps. In the context of individuals with paraplegia, the act of transferring poses a considerable risk, wherein the action of manoeuvring the lower extremities, either before or after a forceful extension from the triceps, exerts substantial biomechanical stress on the spinal architecture.

Significantly, patient 5 exhibited the development of kyphosis, which can be attributed to the progressive degeneration, collapse of vertebral bodies, and weakening of the spinal structures essential for maintaining spinal integrity and alignment. This condition emerges against a backdrop of impaired nociception, wherein the diminished capacity of the body to perceive pain facilitates the continuation of activities devoid of the customary protective pain feedback. Such a scenario predisposes the spinal column to recurrent microtraumas and stress, further exacerbating the progression towards kyphotic deformity. Additionally, neuromuscular scoliosis, as identified in Patient 4, markedly undermined spinal structural integrity, thereby predisposing the patient to the development of CSA.

### Clinical presentation

Symptoms varied among patients but were characteristically non-specific. They included back pain, paradoxical considering the nociceptive deficits, spinal deformity, instability, increased spasticity, and reduced deep tendon reflexes. A critical symptom was autonomic dysreflexia (AD), often triggered by spinal instability, exerting pressure on the presacral plexus or retroperitoneal viscera. The identification and diagnosis of CSA therefore present significant challenges within clinical practice, often resulting in protracted delays. Literature has found the interval between the initial neurological compromise and clinical presentation averages around 15–17 years, with a range extending from 1–42 years [1, 13, 14]. Our case series found the average to be around 37.17 + 11.29 years (range: 20–52). Given the typically prolonged timeline for clinically diagnosing CSA it is crucial for clinicians to maintain awareness of CSA, emphasising the importance of timely diagnosis. Delays in diagnosis can extend the period of management and increase the risk of secondary complications. For instance, advanced stages of kyphosis in patients with CSA have been documented to precipitate the formation of ulcers, which may further progress to develop fistulas bridging the spine and the skin [15]. These fistulae serve as potential foci for infection, a phenomenon substantiated by numerous reports [2]. This underscores the critical need for early detection and intervention in CSA to mitigate the progression to such severe complications.

Clinical diagnosis of CSA is challenging due to its non-specific nature, necessitating reliance on radiographic findings. Diagnostic criteria include impaired proprioception and pain sensation, bony destruction with osteogenesis on imaging, and non-specific chronic inflammation on histology. CSA progresses through an initial atrophic phase, often missed due to subtle imaging findings, however, incidental imaging findings might reveal subtle endplate osteolysis and/or microfractures, as observed in the imaging of Patient 3, followed by a hypertrophic phase characterized by vertebral destruction, osteophytosis, and spondylolisthesis. Transitioning into the hypertrophic phase, the imaging criteria are classically incorporates the “six D’s”: “distension (indicative of soft tissue mass), density (for sclerosis), debris (for bone fragmentation), disorganisation (for joint dislocation), dislocation (for spondylolisthesis), and destruction (for endplate and facet erosions) [16]. ” This framework offers a nuanced and thorough approach for evaluating the progression and severity of CSA, enriching our diagnostic capabilities. MRI and CT imaging play crucial roles in early detection, revealing soft tissue involvement, disc space effusions, and the vacuum phenomenon—a hallmark of CSA, as noted in Patient 1′s imaging [2, 16]. Complementing this, further diagnostic observations encompass advanced degeneration of intervertebral discs, significant erosion of the vertebral body, extensive hypertrophic paravertebral osteophytosis exhibiting a pseudotumoral appearance, and initial deterioration of facet joints, all aligning with the previously mentioned six D’s. The concurrent involvement of all three spinal columns, discernible through radiographic examination, is instrumental in the differential diagnosis process, differentiating CSA from infectious or degenerative conditions [2]. Notably, the pervasive involvement of the vertebral body frequently culminates in spondylolisthesis, as seen in Patient 2.

### Management

Our patients received multidisciplinary treatment involving spinal rehabilitation specialists, surgeons, nurses, physiotherapists, occupational therapists and dieticians. Conservative management, including bed rest, bracing, and physiotherapy, was prioritized for patients without progressive neurological decline or infection. Surgical intervention, specifically circumferential 360° long-segment spinal fusion, constitutes the cornerstone of CSA management, aiming to alleviate back pain, enhance stability, and prevent further neurological impairment, infection, or mortality [17]. This approach is particularly crucial for patients who are ambulatory or exhibit incomplete paraplegia to conserve residual neurological functions. This surgical strategy is advised for patients with complete sensorimotor paraplegia who do not face a risk of further neurological deterioration. Contrarily, the prevailing surgical technique for CSA currently involves circumferential arthrodesis, which employs a combined approach to stabilize both the anterior and posterior vertebral columns [2]. For patients reliant on wheelchairs, maintaining a neutral or slightly flexed sagittal alignment is preferred, enhancing bladder function. The primary goal of surgical intervention is to achieve spinal segment stabilization through high-quality fusion [18]. The surgical protocol often includes debridement of necrotic or inflamed tissue, particularly sclerosed and hypovascularized bone, to create optimal conditions for bone fusion. This debridement process is most effectively performed via an anterior approach. Consensus among researchers underscores the importance of grafting the anterior column, either through an anterior or a posterolateral approach, with the choice of surgical technique largely influenced by the surgeon’s expertise and personal preference [2, 4].

For elderly patients who are not surgical candidates, or for those with minimal neurological deficits, a conservative or non-surgical approach may be deemed appropriate. In instances of CSA characterized by minor bone involvement, the surgical team may opt for a posterior column-only construct, a decision exemplified by the case discussed in patient 1. Postoperative complications, such as implant loosening and additional Charcot lesions, necessitate vigilant long-term surveillance.

### Rehabilitation

Postoperative rehabilitation for CSA is complex, requiring a tailored approach. The case of patient 4 illustrate the multifaceted challenges in recovery, including loss of bowel and bladder control and significant lifestyle adjustments. Rehabilitation involved extensive inpatient care, education, and adaptive processes to manage neurogenic complications and ensure quality of life. The critical role of continuous, multidisciplinary care is emphasized in facilitating recovery and regaining autonomy.

Our patients participated in a comprehensive rehabilitation program tailored to the specific needs of CSA. Daily physiotherapy sessions focused on improving mobility, strength, and functional capacity, with progressive intensification to include gym-based exercises for core stability and postural control—essential for managing CSA’s biomechanical demands. Hydrotherapy was utilized as needed to aid joint mobility and reduce pain while minimizing spinal stress. Weekly occupational therapy sessions aimed to enhance fine motor skills and adapt daily activities to accommodate physical limitations, supporting patient independence in essential tasks. Nutritional support was a critical component, with a dietitian providing ongoing dietary interventions to optimize health, promote tissue healing, and manage pressure ulcers, alongside education on maintaining bone health and preventing further degeneration. Psychological support was also integral, with regular consultations addressing the emotional and psychological challenges of CSA, such as coping with chronic pain and adjusting to functional changes. This holistic approach aimed to build mental resilience and support recovery.

Together, these cases illustrate not just the challenges faced by individuals undergoing surgery for CSA, but also the profound importance of a patient-centered, multidisciplinary approach to rehabilitation. It emphasises comprehensive care, encompassing not only the physical but also the educational, adaptive, and psychosocial dimensions of recovery that can only be achieved within an MDT setting.

There is evidently limited literature on CSA in rehabilitation possibly due to the rarity of the condition, complexity and variability in the presentation making standardised rehabilitation approaches challenging hindering the development of broad guidelines and studies. Most of the existing literature on CSA focuses on surgical management given its role in addressing the structural issue of the spine, yet less attention has been given to the pre-surgical and post-surgical rehabilitation phase.

### Limitations

The limitations of this case series are notably influenced by its retrospective nature, which inherently restricts the accuracy and reliability of data collection, namely selection bias. Furthermore, the extraction of patient data was contingent upon the accuracy of diagnosis codes assigned in their discharge summaries. Should there be discrepancies or inaccuracies in coding, it would preclude the inclusion of such patient data in the extraction process.

Next, given the protracted period over which Charcot joint pathology develops and is subsequently diagnosed, the series spans cases from the 1970s to the early 2000s. This wide temporal range introduces significant variability in the medical management practices, diagnostic criteria, and treatment modalities applied across different periods. Moreover, the evolution of medical technology and the refinement of diagnostic techniques over these decades could further complicate the consistency and reliability of data, potentially impacting the series’ overall findings and conclusions.

## Conclusion

This case series documents six patients, and exemplifies the intricate challenges associated with the long-term management of patients with SCI that developed Charcot spinal arthropathy, a rare but severe complication. It underscores the importance of an integrated, multidisciplinary approach in diagnosing and treating complex spinal disorders, highlighting the role of advanced surgical techniques in mitigating the debilitating effects of such conditions and improving patient outcomes. This endeavour aims to contribute to the broader knowledge base and facilitate a deeper understanding among healthcare professionals, thereby enhancing patient care outcomes in this complex clinical domain.

## References

[1] Lee D, Dahdaleh N. Charcot spinal arthropathy. J Craniovertebr Junction Spine. 2018;9:9. 29755231 10.4103/jcvjs.JCVJS_130_17 PMC5934971

[2] Urits I, Amgalan A, Israel J, Dugay C, Zhao A, Berger AA, et al. A comprehensive review of the treatment and management of Charcot spine. Ther Adv Musculoskelet Dis. 2020;12:1759720X2097949. 10.1177/1759720X20979497 PMC7750571 33414850

[3] Coxon A, Shahid S, Lock A. Estimating the UK population of people living with spinal cord injury. 2024. Available from: https://spinal-research.org/news/every-two-hours-someone-is-paralysed-by-spinal-cord-injury-in-the-uk-new-data-reveals/.

[4] Del Arco Churruca A, Vázquez Bravo JC, Gómez Álvarez S, Muñoz Donat S, Jordá Llona M. Charcot arthropathy in the spine. Experience in our centre. About 13 cases. Review of the literature. Rev Esp Cir Ortop Traumatol (Engl Ed). 2021;65:461–8. 10.1016/j.recot.2020.10.009 34561209

[5] Crim JR, Bassett LW, Gold RH, Mirra JM, Mikulics M, Dawson EG, et al. Spinal neuroarthropathy after traumatic paraplegia. AJNR Am J Neuroradiol. 1988;9:359–62. 3128083 PMC8334244

[6] Devlin VJ, Ogilvie JW, Transfeldt EE, Boachie-Adjei O, Bradford DS. Surgical treatment of neuropathic spinal arthropathy. J Spinal Disord. 1991;4:319–28. 1802163 10.1097/00002517-199109000-00009

[7] Nagasako EM, Oaklander AL, Dworkin RH. Congenital insensitivity to pain: an update. Pain. 2003;101:213–9. 12583863 10.1016/S0304-3959(02)00482-7

[8] Staudt MD, Bailey CS, Siddiqi F. Charcot spinal arthropathy in patients with congenital insensitivity to pain: a report of two cases and review of the literature. Neurosurg Rev. 2018;41:899–908. 28124176 10.1007/s10143-017-0814-3

[9] Cutting PEJ. A case of Charcot’s disease of the cervical spine. BMJ. 1949;1:311–311. 18109341 10.1136/bmj.1.4598.311 PMC2049381

[10] Vialle R, Mary P, Tassin JL, Parker F, Guillaumat M. Charcot’s disease of the spine. Spine. 2005;30:E315–22. 15928542 10.1097/01.brs.0000164283.01454.9f

[11] Aly T. Back muscles injury during posterior lumbar spine surgeries: minimally invasive versus open approaches—a review of the literature. Spine J. 2022;41:61–72.

[12] Dardari D. An overview of Charcot’s neuroarthropathy. J Clin Transl Endocrinol. 2020;22:100239. 33251117 10.1016/j.jcte.2020.100239 PMC7677697

[13] Morita M, Miyauchi A, Okuda S, Oda T, Yamamoto T, Iwasaki M. Charcot spinal disease after spinal cord injury. J Neurosurg Spine. 2008;9:419–26. 18976172 10.3171/SPI.2008.9.11.419

[14] Barrey C, Massourides H, Cotton F, Perrin G, Rode G. Charcot spine: two new case reports and a systematic review of 109 clinical cases from the literature. Ann Phys Rehabil Med. 2010;53:200–20. 20338837 10.1016/j.rehab.2009.11.008

[15] Brown CW, Jones B, Donaldson DH, Akmakjian J, Brugman JL. Neuropathic (Charcot) arthropathy of the spine after traumatic spinal paraplegia. Spine. 1992;17:S103–8. 1631708 10.1097/00007632-199206001-00007

[16] Boudabbous S, Paulin EN, Delattre BMA, Hamard M, Vargas MI. Spinal disorders mimicking infection. Insights Imaging. 2021;12:176. 34862958 10.1186/s13244-021-01103-5 PMC8643376

[17] Moreau S, Lonjon G, Jameson R, Judet T, Garreau de Loubresse C. Do all Charcot spine require surgery?. Orthop Traumatol Surg Res. 2014;100:779–84. 25257755 10.1016/j.otsr.2014.05.021

[18] Von Glinski A, Frieler S, Elia CJ, Ansari D, Pierre C, Ishak B, et al. Surgical management of Charcot spinal arthropathy in the face of possible infection. Int J Spine Surg. 2021;15:752–62. 34315758 10.14444/8097 PMC8375706

